# Body Mass Index a Forecast of Sputum Culture Conversion Among Drug-Resistant Tuberculosis Patients

**DOI:** 10.7759/cureus.63262

**Published:** 2024-06-27

**Authors:** Amaan Latif, R.A.S. Kushwaha, Gaurav Srivastava, Ankit Kumar, Surya Kant, Satish Kumar

**Affiliations:** 1 Respiratory Medicine, Ganesh Shankar Vidyarthi Memorial (GSVM) Medical College, Kanpur, IND; 2 Respiratory Medicine, King George's Medical University, Lucknow, IND; 3 Medicine, King George's Medical University, Lucknow, IND

**Keywords:** outcome research, multidrug resistance tuberculosis, multidrug-resistant (mdr) tb, anti-tuberculosis treatment, body mass index: bmi

## Abstract

Background

Drug-resistant tuberculosis is a major health issue around the world. The time it takes to find a sputum-positive patient is a major risk factor for the spread of tuberculosis, and many things can indicate a longer time to culture conversion. Also, there is strong proof that poor nutrition is linked to infectious diseases. So, this study aimed to look into the link between a person's body mass index (BMI) and the change of a sputum culture within three months in people who have rifampicin-resistant (RR)/multidrug-resistant* (*MDR)-tuberculosis (TB) kept on a bedaquiline-based regimen.

Materials and methods

The Department of Respiratory Medicine at King George's Medical University, Lucknow, hosted an observational, analytical, prospective, single-center study from May 2020 to April 2021. The study included 105 people who had been identified with RR/MDR-TB and were on an optimized background regimen that included a bedaquiline-based regimen. The result we were interested in was sputum culture conversion within three months, and we looked at how BMI related to that outcome. Analytical analyses utilized Pearson’s chi-square test for categorical variables and the t-test for continuous variables. Differences with a P-value of <0.05 were considered significant. SPSS software (version 18.0, IBM Corp., Armonk, NY, USA) was used for all analyses, with missing data not replaced or credited.

Results

A total of 105 people who met the inclusion and exclusion criteria were analyzed. The patients had a mean age of 33.34 years and were mostly male 61 (58%). Fifty-eight (58; 55%) patients lived in rural areas. Most patients had fever 77 (73%), cough 72 (69%), and weight loss 66 (63%). Sixty-nine (69; 66%) patients had a history of TB. Fifty-seven patients had a BMI of <18.5 kg/m^2^, and 48 patients had with BMI of ≥18.5. At the end of the study, 75/105 patients converted their sputum culture. Of the 105 patients, 57 (54%) had a low BMI (less than 18.5 kg/m^2^). Among the 57 patients with a BMI of <18.5 kg/m^2^, only 28 (46%) achieved sputum culture conversion after 3 months while 29 (60%) of 48 with BMI ≥18.5 achieved sputum culture conversion after 3 months. Among the patients with a BMI <18.5, 15/57 (26%) tested positive for sputum culture after three months. In patients with a BMI of ≥18.5, only 4/48 (8%) patients tested positive for sputum culture after three months.

Conclusion

In patients with drug-resistant tuberculosis, low BMI (<18.5 kg/m^2^) was an independent risk factor for failing to convert sputum cultures within three months.

## Introduction

Tuberculosis (TB) is one of the most persistent health problems in the world with a history dating back nearly 10,000 years [1]. TB claims the lives of millions each year. This disease disproportionately affects socioeconomically disadvantaged populations, particularly in low and middle-income countries across Asia and Africa [2].

India has a high TB burden, contributing to a quarter of all new cases reported globally each year. Within this context, India also faces a substantial challenge in combating drug-resistant forms of TB, including multidrug-resistant tuberculosis (MDR-TB) and rifampicin-resistant tuberculosis (RR-TB). Recent estimates reveal many new RR/MDR-TB cases [3,4].

Findings from the first national anti-tuberculosis drug resistance survey (NDRS) revealed alarmingly high rates of drug resistance among TB patients in India. This high burden underscores the gravity of the situation. Particularly those who have previously received treatment and exhibit resistance to first-line drugs such as isoniazid and rifampicin. This means that these patients need more complicated and resource-intensive treatments [5].

Bedaquiline is a primary drug for the treatment of multidrug-resistant tuberculosis (MDR-TB). It works by inhibiting the mycobacterial ATP synthase. This enzyme is essential for the energy production of Mycobacterium tuberculosis. Bedaquiline is usually administered in combination with other anti-TB drugs to enhance its efficacy and prevent the development of further drug resistance. A bedaquiline-based regimen is part of a broader treatment plan that can last 18-20 months. The duration of therapy depends on the patient's response and the presence of other drug resistances. This regimen has been associated with higher rates of sputum culture conversion and lower mortality compared to regimens without bedaquiline [3].

One of the critical challenges in TB management is ensuring successful treatment outcomes, which indicates treatment efficacy and reduced infectiousness. Among drug-resistant patients, timely culture conversion poses a significant hurdle. Poor adherence to treatment regimens is a common issue, leading to treatment failure and the emergence of MDR-TB strains with additional resistance [3,6,7].

Given these challenges, there is a growing interest in investigating adjunctive factors that may influence treatment outcomes among patients with drug-resistant TB. Body mass index (BMI) has emerged as a potential prognostic indicator for sputum culture conversion among DR-TB patients. Understanding the relationship between BMI and treatment outcomes could offer valuable insights into optimizing TB management strategies and improving patient care.

## Materials and methods

Study population

Ethical clearance was taken before the initiation of the study. This observational, analytical, prospective, single-center study included patients treated for MDR-TB between May 2020 and April 2021. Suspected MDR-TB cases referred to the MDR-TB clinic at Respiratory Medicine, King George's Medical University, Lucknow, were evaluated. Patient records were reviewed for demographic data, TB treatment history, chest radiographs, comorbidities, drug susceptibility testing (DST) results, and clinical outcomes. Follow-up was conducted until completion of treatment or loss of follow-up.

Patients aged 18 years or older of both sexes were eligible for inclusion in the study. People who had been diagnosed with DR-TB and were being treated with an optimized bedaquiline-based regimen were included. Informed consent was a prerequisite for inclusion in the study.

Patients who failed to provide informed consent or became lost to follow-up were excluded from the study. Additionally, individuals with extrapulmonary tuberculosis or those not receiving a bedaquiline-based optimized regimen for DR-TB pulmonary tuberculosis were ineligible for participation. Patients were excluded if height and weight data were unavailable.

From the third to sixth months of treatment, quarterly assessments followed by monthly culture reports made up the evaluation process. We rigorously assessed compliance with the treatment regimen during these intervals. Patients lost to follow-up after enrolment, totaling five individuals, were subsequently excluded from the analysis.

At the end of the study, all 105 patients who had been enrolled were re-evaluated to find out how well their treatment worked and what effect BMI had on the conversion of sputum cultures in the management of MDR-TB.

Sputum cultures and DST

The MDR-TB diagnosis relied on sputum cultures and DST results. We performed cultures using the MGIT960 liquid culture system and conducted DST using Lowenstein-Jensen agar slants and multiple drugs. Drug resistance was defined as a growth rate of >1% compared to the control. Sputum culture conversion was defined as two consecutive negative cultures at least 30 days apart, with time to conversion calculated from treatment initiation.

Treatment

We treated patients according to programmatic management of drug-resistant tuberculosis (PMDT) guidelines, considering their DST results and treatment history. Treatment regimens consisted of at least five targeted drugs, modified as necessary by TB specialists. Follow-up continued after discharge, with treatment duration ranging from 18 to 20 months based on culture results, radiographic changes, and treatment history.

Definition

Pre-treatment BMI was calculated as weight divided by height squared (kg/m^2^). Patients were categorized as underweight, normal weight, overweight, or obese based on BMI values. The outcome of interest was sputum culture conversion within three months, and the association between BMI and this outcome was analyzed.

Data analysis

Descriptive analyses utilized Pearson’s chi-square test for categorical variables and the t-test for continuous variables. Differences with a P-value of <0.05 were considered significant. SPSS software (version 18.0, IBM Corp., Armonk, NY, USA) was used for all analyses, with missing data not replaced or credited.

## Results

Of 110 patients with DR-TB, 105 who met the inclusion and exclusion criteria were analyzed. The patients had a mean age of 33.34 years and were mostly male (61; 58%). Fifty-eight (58; 55%) patients lived in rural areas and had a mean BMI of 18.7 ± 1.15 kg/m^2^. Most patients had fever 77 (73%), cough 72 (69%), and weight loss 66 (63%). Sixty-nine (69; 66%) patients had a history of TB and had previously received treatment. At the end of the study, a total of 75/105 patients converted their sputum culture (culture negative for Mycobacterium tuberculosis). Out of these 105 patients, 57 had a BMI of <18.5 kg/m^2,^ and 48 had a BMI of >18.5 kg/m^2^.

Among the patients with a BMI of <18.5 kg/m^2^, only 28 (46%) patients achieved sputum culture conversion after three months while 29 (60%) patients of the 48 with a BMI of ≥18.5 achieved sputum culture conversion after three months. Similarly, in patients with a BMI of <18.5, 15/57 (26%) patients tested positive for sputum culture after three months. In patients with a BMI of ≥18.5, only 4 of 48 patients tested positive for sputum culture after three months (P-value 0.033). Few culture reports are unavailable or contaminated due to the difficulty of patient follow-up during the COVID era (Table 1). In the study population, 20 patients expired, out of which 14 expired in patients with a BMI of <18.5 while only 6 expired in patients with a BMI of ≥18.5. In our study, 29 patients had unfavorable outcomes, comprising treatment failure, culture reversion, and expiry. Out of those 29 patients, 19 (65%) had a lower BMI of <18.5. In patients with a BMI of >18.5, only 10 patients had unfavorable outcomes, comprising treatment failure, culture reversion, and expiry (Table 2).

**Table 1:** Body mass index cross-tabulation with the third-month smear result M3=third month, C=contamination, N=negative, and P=positive Data were presented in numbers and percentages within the body mass index and culture conversion at the third month; differences with a P-value of <0.05 were considered significant.

BMI * M3 cross-tabulation							
			M3				Total
			Not Available	C	N	P	
BMI	Underweight	Count	14	2	26	15	57
		% within BMI	24.6%	3.5%	45.6%	26.3%	100.0%
		% within M3	53.8%	40.0%	47.3%	78.9%	54.3%
		% of Total	13.3%	1.9%	24.8%	14.3%	54.3%
	Normal weight	Count	12	3	23	4	42
		% within BMI	28.6%	7.1%	54.8%	9.5%	100.0%
		% within M3	46.2%	60.0%	41.8%	21.1%	40.0%
		% of Total	11.4%	2.9%	21.9%	3.8%	40.0%
	Overweight	Count	0	0	5	0	5
		% within BMI	0.0%	0.0%	100.0%	0.0%	100.0%
		% within M3	0.0%	0.0%	9.1%	0.0%	4.8%
		% of Total	0.0%	0.0%	4.8%	0.0%	4.8%
	Obesity class 1	Count	0	0	1	0	1
		% within BMI	0.0%	0.0%	100.0%	0.0%	100.0%
		% within M3	0.0%	0.0%	1.8%	0.0%	1.0%
		% of Total	0.0%	0.0%	1.0%	0.0%	1.0%
Total		Count	26	5	55	19	105
		% within BMI	24.8%	4.8%	52.4%	18.1%	100.0%
		% within M3	100.0%	100.0%	100.0%	100.0%	100.0%
		% of Total	24.8%	4.8%	52.4%	18.1%	100.0%
						The chi-square test for sputum-positive rates after three months between the underweight group vs all other BMI categories shows chi-square value: 4.537, p-value: 0.033	

**Table 2:** Body mass index cross tabulation with final outcome BMI=body mass index, OUTC=outcome Data were presented in numbers and percentages within body mass index and culture conversion at the third month; differences with a P-value of <0.05 were considered significant.

BMI * OUTCOME Cross-tabulation								
			OUTCOME					Total
			culture conversion	culture reversion	cured	expired	failure	
BMI	Underweight	Count	26	1	12	14	4	57
		% within BMI	45.6%	1.8%	21.1%	24.6%	7.0%	100.0%
		% within OUTC	51.0%	50.0%	48.0%	70.0%	57.1%	54.3%
		% of Total	24.8%	1.0%	11.4%	13.3%	3.8%	54.3%
	Normal weight	Count	23	0	10	6	3	42
		% within BMI	54.8%	0.0%	23.8%	14.3%	7.1%	100.0%
		% within OUTC	45.1%	0.0%	40.0%	30.0%	42.9%	40.0%
		% of Total	21.9%	0.0%	9.5%	5.7%	2.9%	40.0%
	Overweight	Count	2	1	2	0	0	5
		% within BMI	40.0%	20.0%	40.0%	0.0%	0.0%	100.0%
		% within OUTC	3.9%	50.0%	8.0%	0.0%	0.0%	4.8%
		% of Total	1.9%	1.0%	1.9%	0.0%	0.0%	4.8%
	Obesity class 1	Count	0	0	1	0	0	1
		% within BMI	0.0%	0.0%	100.0%	0.0%	0.0%	100.0%
		% within OUTC	0.0%	0.0%	4.0%	0.0%	0.0%	1.0%
		% of Total	0.0%	0.0%	1.0%	0.0%	0.0%	1.0%
Total		Count	51	2	25	20	7	105
		% within BMI	48.6%	1.9%	23.8%	19.0%	6.7%	100.0%
		% within OUTC	100.0%	100.0%	100.0%	100.0%	100.0%	100.0%
		% of Total	48.6%	1.9%	23.8%	19.0%	6.7%	100.0%

## Discussion

When you look at our study along with other research, you can learn a lot about how BMI affects treatment outcomes.

This study involved 105 drug-resistant tuberculosis (DR-TB) patients, with a median age of 33.34 (SD 14.1) years. The age distribution revealed that the incidence of DR-TB was highest among patients aged 18-30 years, followed by the 30-40 age group. The higher incidence of resistance in younger patients might be attributed to their reluctance to adhere to prescribed medication regimens. In support of this, a study by Abdulhalik Workicho et al. found that among different sociodemographic factors, respondents' age was strongly linked to the presence of MDR-TB. People younger than 30 years old were seven times more likely to have MDR-TB than people older than 30 years old [8].

According to the gender distribution table, pulmonary tuberculosis cases are more prevalent among males as compared to females. This disparity can be attributed to the infectious nature of pulmonary TB and the generally lower health-seeking behavior observed in females, leading to fewer reported cases among women. A similar trend was observed in a meta-analysis by Pradipta et al., which revealed that males are more prone to developing multidrug-resistant tuberculosis (MDR-TB) than females. This finding underscores the importance of addressing gender-specific barriers to healthcare access and TB treatment adherence [9].

The majority of patients were underweight (57; 54%) and 42 (40%) were normal weight (Table 1). Undernourishment is a known risk factor for tuberculosis. The study by Lin et al. found that higher BMI levels were inversely associated with tuberculosis risk, with obese individuals having a two-thirds reduction in risk compared to normal-weight individuals [10].

Kwon et al. (2008) assessed treatment outcomes for HIV-uninfected patients with MDR-TB, highlighting the complexity of managing this condition and the importance of optimizing treatment regimens to improve outcomes [11]. Our study adds to this body of evidence by identifying low BMI as an independent risk factor for poor treatment response, complementing the understanding of factors influencing MDR-TB outcomes.

Park et al. (2016) investigated the association between BMI and sputum culture conversion in South Korean patients with MDR-TB [12]. Their findings align with ours, emphasizing the impact of low BMI on treatment response. This consistency across different populations strengthens the evidence for considering BMI as a prognostic factor in MDR-TB management [12].

Cegielski and McMurray (2004) reviewed the relationship between malnutrition and tuberculosis, emphasizing the bidirectional impact of these conditions [13]. Our study contributes to this understanding by specifically examining the role of BMI in MDR-TB treatment outcomes, providing insights into the implications of undernutrition in this context.

Putri et al. investigated how well BMI could predict sputum culture conversion in MDR-TB patients in Indonesia. Their results supported ours about the link between low BMI and treatment response. This consistency across diverse populations underscores the robustness of BMI as a prognostic indicator in MDR-TB [14].

Bhargava et al. (2014) estimated the population-attributable fraction of TB cases related to undernutrition in India, underscoring the significance of nutritional interventions in TB prevention and control [15]. Our study builds on this by specifically examining the impact of BMI on treatment outcomes in MDR-TB patients, providing clinical evidence for the role of nutrition in MDR-TB management.

Diallo et al. (2020) identified different BMI profiles among patients with MDR-TB, highlighting the heterogeneity in treatment responses and emphasizing the need for personalized approaches in TB management [16]. Our study contributes to this understanding by elucidating the association between BMI and treatment outcomes, further supporting the importance of individualized care in MDR-TB.

Our study offers several notable contributions to MDR-TB management. By focusing on BMI's impact on sputum culture conversion in MDR-TB patients, we provide actionable insights directly relevant to clinical practice. Moreover, our identification of low BMI as an independent risk factor underscores the need for tailored interventions to improve treatment efficacy.

While conducted at a single tertiary TB referral center, our study's diverse patient population enhances its generalizability to similar clinical settings. However, limitations, such as the relatively small sample size and exclusion criteria, warrant caution in extrapolating findings. We need larger-scale, multi-center studies to validate results and address potential biases introduced by the COVID-19 pandemic.

The recognition of low BMI as a predictor of poor treatment response underscores the importance of nutritional assessment and support in MDR-TB management. Integrating BMI monitoring into treatment protocols can facilitate early intervention and personalized care, ultimately improving patient outcomes.

Moving forward, research efforts should focus on expanding sample sizes and conducting longitudinal studies to further elucidate the relationship between BMI and treatment outcomes in MDR-TB. Additionally, exploring the efficacy of nutritional interventions alongside standard treatments could enhance therapeutic success and reduce the burden of MDR-TB on global health.

## Conclusions

Our study contributes to the existing literature by highlighting the association between low BMI and poor treatment response in MDR-TB patients. By putting our results in the bigger picture of tuberculosis research, we show how important it is to take nutritional factors into account when treating MDR-TB and work on personalized methods to make treatment work better.
